# Improved Stability of Emulsions in Preparation of Uniform Small-Sized Konjac Glucomanna (KGM) Microspheres with Epoxy-Based Polymer Membrane by Premix Membrane Emulsification

**DOI:** 10.3390/polym8030053

**Published:** 2016-02-23

**Authors:** Yace Mi, Juan Li, Weiqing Zhou, Rongyue Zhang, Guanghui Ma, Zhiguo Su

**Affiliations:** 1 National Key Laboratory of Biochemical Engineering , Institute of Process Engineering, Chinese Academy of Sciences, Beijing 100190, China; miyacetx@163.com (Y.M.); lijuan@ipe.ac.cn (J.L.); wqzhou@ipe.ac.cn (W.Z.); ryzhang@iccas.ac.cn (R.Z.); zgsu@ipe.ac.cn (Z.S.); 2 University of Chinese Academy of Sciences, Beijing 100049, China

**Keywords:** uniform small-sized KGM microspheres, premix membrane emulsification, Epoxy-based polymer membrane (EP), alkaline condition

## Abstract

Uniform small-sized (<10 μm) Konjac glucomanna (KGM) microspheres have great application prospects in bio-separation, drug delivery and controlled release. Premix membrane emulsification is an effective method to prepare uniform small-sized KGM microspheres. However, since KGM solution bears strong alkalinity, it requires the membrane to have a hydrophobic surface resistant to alkali. In this study, uniform small-sized KGM microspheres were prepared with epoxy-based polymer membrane (EP) we developed by premix membrane emulsification. It was found that emulsion coalescence and flocculation occurred frequently due to the high interface energy and sedimentation velocity of KGM emulsions. Emulsion stability had a significant influence on the uniformity and dispersity of the final KGM microspheres. To improve the stability of the emulsions, the effects of the concentration of the emulsifier, the viscosity of the KGM solution, the oil phase composition and the feeding method of epoxy chloropropane (EC) on the preparation results were studied. Under optimal preparation conditions (emulsifier 5 wt % PO-5s, KGM III (145.6 mPa·s), weight ratio of liquid paraffin (LP) to petroleum ether (PE) 11:1), uniform and stable KGM emulsions (*d* = 7.47 μm, *CV* = 15.35%) were obtained and crosslinked without emulsion-instable phenomena.

## 1. Introduction

Konjac glucomannan (KGM), as a high-molecular-weight polysaccharide, has special physical characteristics such as moisture retentivity, thickening, and gelling properties [1] and bears so many hydroxyl groups that various chemical modifications can be realized on KGM molecular chains [2,3,4,5,6]. KGM microspheres based on KGM powder have great application prospects in the fields of separation and purification [7], cell culture [8], enzyme and cell immobilization [9], drug delivery [10] and controlled release [11]. At present, the research on the preparation of KGM microspheres is insufficient, especially on those with small particle size (<10 μm). The most commonly used method to prepare KGM microspheres is mechanical stirring. In our previous work [12], a technology was developed to prepare KGM microspheres, which included acid degradation, alkali dissolution of KGM powder, emulsion preparation by mechanical stirring, and crosslinking steps. Compared with the traditional method, this method greatly shortened the reaction time and simplified the preparation process. However, since these microspheres prepared by this method were not uniform, sieving was necessary, and this resulted in higher product costs. It was more important to discover that uniform microspheres with small particle size (<10 μm) cannot be prepared by this method. Jianhua Shen [13] has reported a method of injection with template. It was characterized by the use of template to form homogeneous emulsions with controllable particle size. However, the size of the KGM microspheres prepared by this method was in the range of 50–500 μm, and small-sized (<10 μm) microspheres cannot be obtained.

Membrane emulsification technology [14], as a novel emulsion preparation method, has huge application prospects [15]. Different from the traditional technology, membrane emulsification can be used to produce uniform emulsions with a particle size less than 10 μm [16]. In this study, this technology was chosen to prepare small-sized KGM microspheres. KGM solutions used for the preparation of KGM microspheres bear strong alkalinity [17], which requires the membrane to have a hydrophobic surface resistant to alkali. However, Shirasu Porous Glass (SPG) membrane (Ise Chemical Co., Ise, Japan), as the most commonly used membrane in membrane emulsification [18], is chemically unstable in alkaline solution [19]. The main component of SPG is SiO_2_ which is not resistant to alkali. If used in alkaline conditions chronically, SPG will become semitransparent, which means some pores in SPG have collapsed [20]. Furthermore, SPG is hydrophilic in nature due to a great amount of Si–OH groups on its surface [21]. Hydrophobic modification of SPG is needed in the preparation of water-in-oil (W/O) emulsions [22]. However, the hydrophobic ligands on the surface of SPG come off easily under alkaline conditions [19]. Therefore, SPG membrane cannot be directly used in the preparation of KGM microspheres. In a previous work, we obtained a kind of epoxy-based polymer (EP) membrane with a three-dimensional bicontinuous structure and stable hydrophobic surface [23]. With the appropriate porosity and narrow pore size distribution, as well as special pore structure, the EP membrane has been successfully applied in membrane emulsification to prepare W/O emulsions. It was worth noting that EP membrane can be applied in alkali systems due to its resistance to alkali [20]. In this study, uniform KGM emulsions were firstly prepared with EP membrane by premix membrane emulsification. 

In the preparation process of KGM microspheres, emulsion coalescence and flocculation occur due to the high interface energy and sedimentation velocity of small-sized KGM emulsions [24]. Emulsion coalescence between two emulsion droplets is irreversible, and it will decrease the number and increase the size of emulsion droplets. Flocculation is a process where two or more emulsion droplets are adhered together into a whole by collision. These aggregated emulsion droplets will disperse again by shaking. However, if it occurs in the crosslinking process, those flocculated microspheres will be solidified and this flocculation is irreversible [25]. In practical cases, coalescence and flocculation may occur simultaneously. Some measures should be taken to avoid these phenomena and improve the preparation results.

In this study, the EP membrane we developed was firstly used in basic system to prepare uniform KGM microspheres by premix membrane emulsification. The effects of the concentration of emulsifiers, the viscosities of KGM solutions and the oil phase composition on the emulsification and crosslinking results were systematically studied. Meanwhile, different crosslinking results were obtained with the same formula by changing the feeding methods of epoxy chloropropane (EC). 

## 2. Materials and Methods

### 2.1. Materials

Konjac powder (the content of KGM was approximately 65% (*w*/*w*)) was obtained from Hu-Bei Konson Konjac Gum Co. Ltd. (Hubei, China). Hexaglycerinpenta ester (PO-5s) purchased from Sakamoto Yakuhin Kogya Co. Ltd. (Osaka, Japan) was used as emulsifier. Liquid paraffin (LP) and petroleum ether (PE) were chosen as oil phase and were supplied by Sinopharm and Tianjin Jin Dong Tian Zheng Precision Chemical Reagent Factory (Tianjin, China), respectively. Epoxy chloropropane (EC) provided by Xilong Co. Ltd. (Guangdong, China) was used as crosslinking agent. Other reagents were of analytical purity and were purchased from Beijing Chemical Reagent Company (Beijing, China).

### 2.2. Preparation of KGM Solutions and Determination of the Viscosities of KGM Solutions and Oil Phases

**Figure 1:**
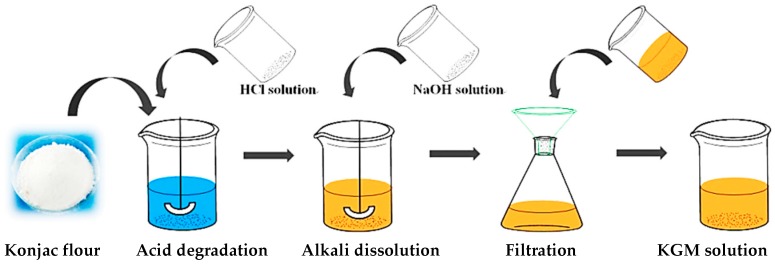
Scheme of the preparation of Konjac glucomanna (KGM) solution.

Since 1% (*w*/*w*) KGM dissolved in water takes on a gelatin texture, it is too viscous to disperse in oil phase. Therefore, it is necessary to degrade the KGM molecules into small ones to improve its flowability. Here, KGM solutions with different viscosities were obtained by acid degradation. The preparation procedure was shown in Figure 1. A certain amount of hydrochloric acid (HCl, 0.5 M) was mixed with deionized water, and then the Konjac powder was added. With enough stirring, the mixture became jelly and then was heated at 115 °C for 55 min. After that, 45% (*w*/*w*) sodium hydroxide was added under vigorous stirring. Finally, 8% (*w*/*w*) KGM solutions with different viscosities were prepared (Table 1).

The viscosities of KGM solutions and oil phases were measured with a Brookfield viscometer (DV-II+Pro) using spindle 62 (50 rpm and 40 °C) and spindle 61 (50 rpm and 25, 40, 50, 60, 70, and 80 °C), respectively.

### 2.3. Preparation of KGM Microspheres by Premix Membrane Emulsification

**Figure 2:**
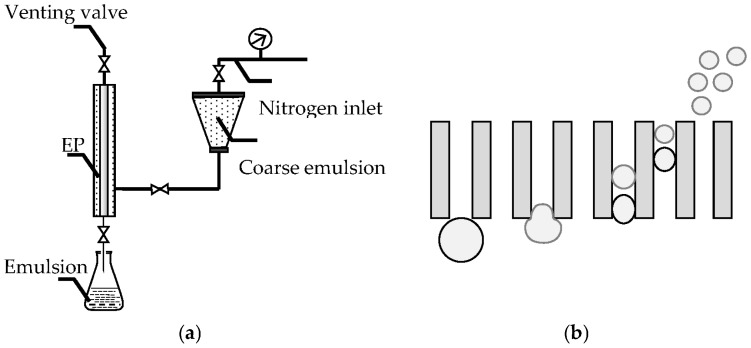
Schematic diagram of premix membrane emulsification apparatus and principle. (**a**) Premix membrane emulsification apparatus; (**b**) Premix membrane emulsification principle.

The schematic diagram of premix membrane emulsification was shown in Figure 2. The coarse emulsions prepared by traditional method were pressed through a membrane with narrow pore size distribution under high pressure. The particle size of the obtained emulsions became smaller and more uniform. The tubular EP membrane used in this study has inside diameter of 0.8 cm, outside diameter of 1.0 cm and length of 2 cm.

The preparation of KGM microspheres by premix membrane emulsification was as follows: the mixture of liquid paraffin and petroleum ether (weight ratio 7:5, 10:2, 11:1, 12:0) with an emulsifier PO-5s (3%, 4%, 5%, 6%) was chosen as oil phase. Aqueous phase of KGM solution (8.0 wt %, 3 mL) was emulsified in oil phase (40 mL) by homogenization (3600 rpm, 1 min) to form coarse emulsion. Then the coarse emulsion was poured into storage tank and pressed through EP membrane five times under nitrogen pressure to achieve uniform emulsions. EC (2 mL) as crosslinking agent was slowly added into the emulsions at 60 °C by burette, and then the mixtures were kept at 60 °C for 8 h to obtain the particles. Finally, the KGM particles were collected and successively washed by petroleum, ethanol and water. The pore sizes of EP membranes were 14.80 μm with operating pressure of 85 kPa. Recipe for the preparation of KGM microspheres was shown in Table 2.

### 2.4. Determination of the Size and Size Distribution of KGM Emulsions

The average KGM emulsions size and size distribution coefficient of variation (*CV*) were determined by automatic analysis software on the basis of optical photographs taken by optical microscope (XSZ-H3). Diameters of over 500 droplets were analyzed to calculate the average particle size and particle size coefficient variation according to Equations (1) and (2). Wherein, *d*_i_ was the diameter of a single microsphere, $\bar{d}$ was the number-average particle diameter.

(1) $$C.V.={\left(\sum_{i=1}^{n}\frac{{\left(d_{i}-\bar{d}\right)}^{2}}{N}\right)}^{\frac{1}{2}}/\bar{d}$$

(2) $$\bar{d}=\sum_{i=1}^{n}d_{i}/N$$

### 2.5. Determination of Interfacial Tension between Aqueous and Oil Phases

The interfacial tension between aqueous and oil phases was determined by Contact angle system OCA (Dataphysics, Berlin, Germany). The aqueous phase was injected into the oil phase through the pinhole under the action of the microflow pump. This process would be recorded by video imaging system and then the interface tension was calculated through the video.

## 3. Results

In the preparation process of KGM microspheres with small particle size, emulsion coalescence and flocculation occurred due to the high interface energy and sedimentation velocity of KGM emulsions. Emulsion stability had a significant influence on the uniformity and dispersity of the final KGM microspheres. To improve the stability of emulsions, the effects of the concentration of the emulsifier, the viscosity of the KGM solution, the oil phase composition and the feeding method of EC on the preparation of KGM microspheres were studied.

### 3.1. Effects of Emulsifier Concentration on the Preparation of KGM Microspheres

Emulsifier adsorbed on the interface between oil and water could improve the stability of emulsions by forming a layer of viscoelastic membrane with a certain strength [26]. The emulsifier concentration has an important effect on the performance of membrane emulsification. After preliminary investigation, PO-5s was chosen as an emulsifier and the effects of its concentration on the preparation of KGM microspheres were investigated. Preparation parameters are shown in Table 2 (**S1**, **S2**, **S3**, **S4**).

**Figure 3:**
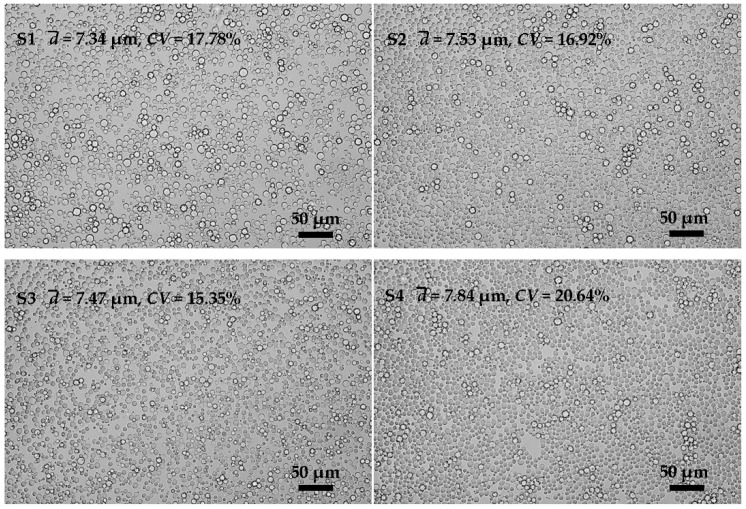
Microscopic photographs of KGM emulsions prepared with different emulsifier concentration (3%, 4%, 5% and 6%, Po-5S, *w*/*w*).

The emulsification results are found in Figure 3 (**S1**, **S2**, **S3**, and **S4**). It shows that when the emulsifier concentration was increased from 3 wt % to 5 wt %, both the *CV* values and particle sizes of the KGM emulsions changed little. When the emulsifier concentration reached 6%, the *CV* value (20.64%) and the particle size (7.84 μm) had marked changes. The effects of the emulsifier concentration were not obvious until it was seriously excessive. The emulsifier played two main roles in the formation of the emulsions. One was lowering the interfacial tension between the oil and water, and the other one was stabilizing the emulsion droplets against coalescence and/or flocculation [27]. Increasing the amount of emulsifier to a proper amount could improve emulsion stability. However, when the emulsifier was excessive, it would play the opposite role [28]. The excess emulsifier will not further reduce the interfacial energy; instead, it will adsorb on the surface of the membrane pores. The more emulsifier was added, the more likely the EP membrane was covered with emulsifier following when it was wetted by the aqueous phase in the emulsification process. In this process, large-size droplets would be produced. 

**Figure 4:**
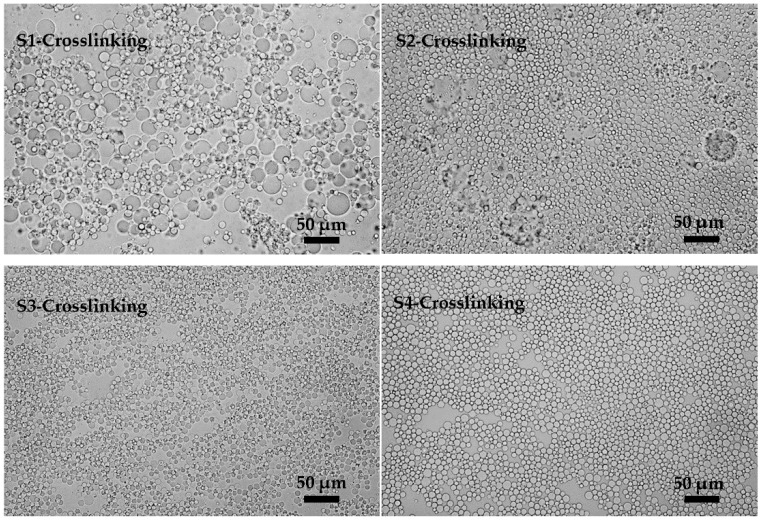
Microscopic photographs of KGM microspheres prepared with different emulsifier concentrations (3%, 4%, 5% and 6%, Po-5S, *w*/*w*): S1-Crosslinking, S2-Crosslinking, S3-Crosslinking and S4-Crosslinking were the crosslinking results of **S1**, **S2**, **S3** and **S4**.

In the following crosslinking process of the KGM emulsions, the effects brought by the changes of emulsifier concentration cannot be ignored. The crosslinking results are shown in Figure 4 (S1-Crosslinking, S2-Crosslinking, S3-Crosslinking, and S4-Crosslinking). It was found that flocculation and coalescence occurred in S1-Crosslinking. Meanwhile, there were two sizes of particles which conglutinated to the gobbet. With the increase of emulsifier concentration, the phenomena of flocculation and coalescence were weakened and finally disappeared when the emulsifier concentration reached 5%. Emulsifier was the key factor in keeping the stability of the emulsions. Emulsions a with low content of emulsifier were unstable due to the high interface energy [29]. Increasing the emulsifier concentration would reduce the interfacial energy and improve the stability of the emulsions against the occurrence of flocculation and coalescence. In summary, the stability of the emulsions was important for the crosslinking results and 5% (*w*/*w*) was the optimal emulsifier concentration in terms of the uniformity and stability of emulsions.

### 3.2. Effects of the Viscosity of KGM Solution on the Preparation of KGM Microspheres

The content of KGM solution has great significance for the application of KGM microspheres. Since viscosities were the main difference in KGM solutions with different contents, the effects of the viscosity of KGM solution on the preparation of microspheres were studied. By changing the amount of acid in the process of acid degradation, four kinds of KGM solutions (Table 1) with different viscosities (66.0, 88.4, 145.6 and 180.3 mPa·s) were obtained.

**Figure 5:**
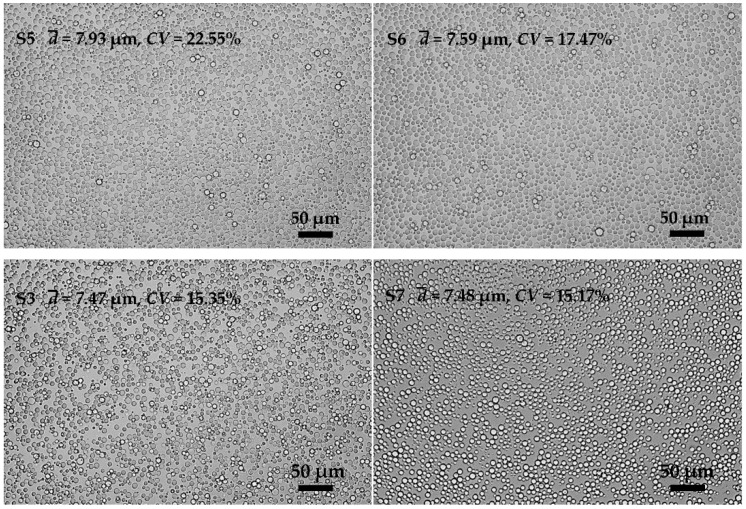
Microscopic photographs of KGM emulsions prepared with different KGM viscosities (KGM I, KGM II, KGM III and KGM IV).

KGM emulsions prepared with different viscosities of KGM solutions as aqueous phase are shown in Figure 5 (**S5**, **S6**, **S3**, and **S7**). The emulsification results varied little in the particle size and uniformity of emulsions, except for the emulsions prepared with a low viscosity of KGM solutions (Figure 5 (**S5**)), the uniformity of which was relatively poor (*CV* = 22.55%). Coalescence could occur in the emulsification process due to the low viscosity of the aqueous phase, which would decrease the uniformity of the final emulsions. Therefore, high viscosity of aqueous phase showed advantages for obtaining uniform emulsions.

**Figure 6:**
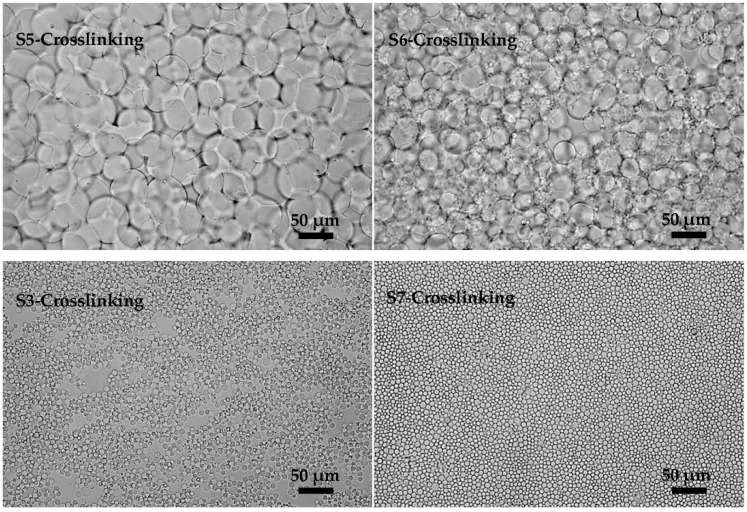
Microscopic photographs of KGM microspheres prepared with different KGM viscosities (KGM I, KGM II, KGM III and KGM IV): S5-Crosslinking, S6-Crosslinking, S3-Crosslinking and S7-Crosslinking were the crosslinking results of **S5**, **S6**, **S3** and **S7**.

Figure 6 (S5-Crosslinking, S6-Crosslinking, S3-Crosslinking, and S7-Crosslinking) shows the crosslinking results. With a low viscosity of the KGM solution (Figure 6 (S5-Crosslinking)), the final microspheres had a much larger size compared with those before crosslinking. These large microspheres adhered to each other. By increasing the viscosity of KGM solution (Figure 6 (S6-Crosslinking)), two sizes of microspheres were produced. Compared with those in **S5**, the flocculation and coalescence were apparently weakened. The dispersity and uniformity of the final microspheres got much better after a further increase of the viscosity of the KGM solution (Figure 6 (S6-Crosslinking and S7-Crosslinking)). Low viscosity of the KGM solution possessed good liquidity contributing to mass transfer, which would induce coalescence in the collision process between emulsion droplets. In addition, low viscosity of KGM solution had low viscoelasticity, which would cause flocculation [30]. In the viscosity range we have studied, the KGM microspheres prepared with high viscosity of aqueous phases had good uniformity and dispersity. The greater the viscosity of the KGM solution, the higher the transmembrane pressure needed in premix membrane emulsification was. So considering the transmembrane pressure, we chose KGM III as the optimal aqueous phase.

### 3.3. Effects of Oil Phase Composition on the Preparation of KGM Microspheres

**Figure 7:**
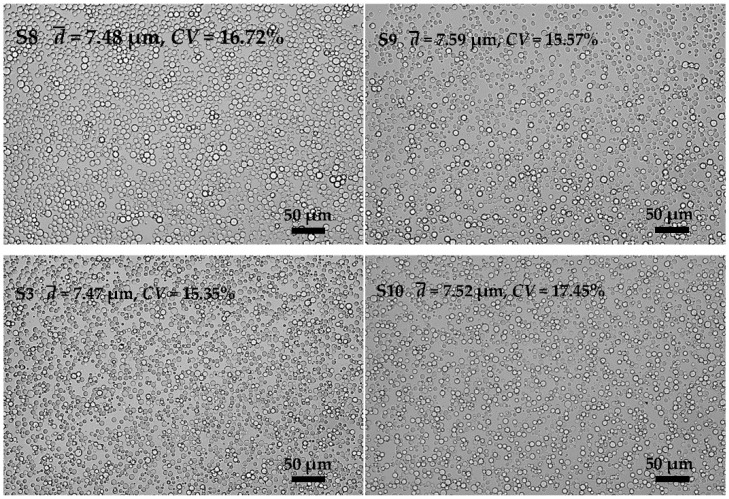
Microscopic photographs of KGM emulsions prepared with different oil phase compositions (7:5, 10:2, 11:1 and 12:0, LP/EP, *w*/*w*).

As one of the major components of emulsions, the continuous phase has a decisive influence on the stability of emulsions. We investigated the effects of oil phase composition in the following work. Figure 7 (**S8**, **S9**, **S3**, and **S10**) shows the preparation results of KGM emulsions by premix membrane emulsification. We found that the oil phase composition had little effect on the droplet size and size uniformity of the emulsions. However, the changes of the oil phase composition had considerable influence in the crosslinking process. 

**Figure 8:**
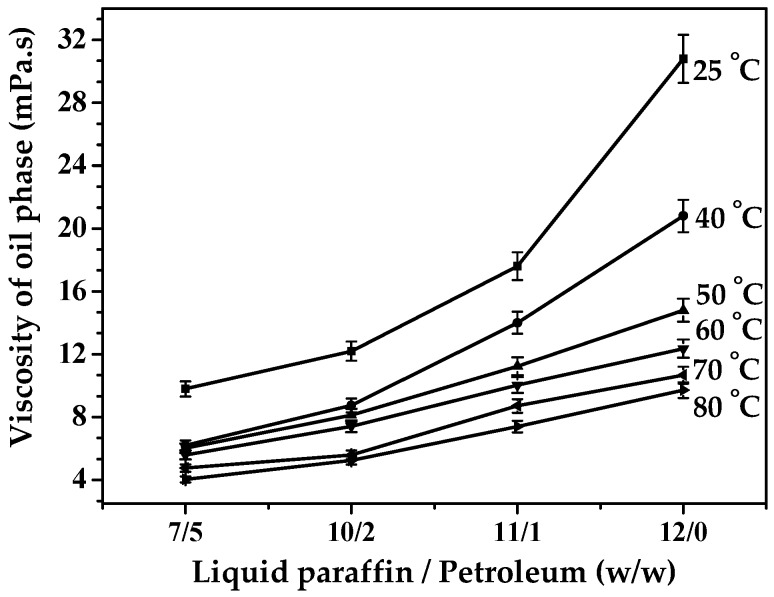
Viscosities of oil phase with different weight ratios of liquid paraffin to petroleum ether.

**Figure 9:**
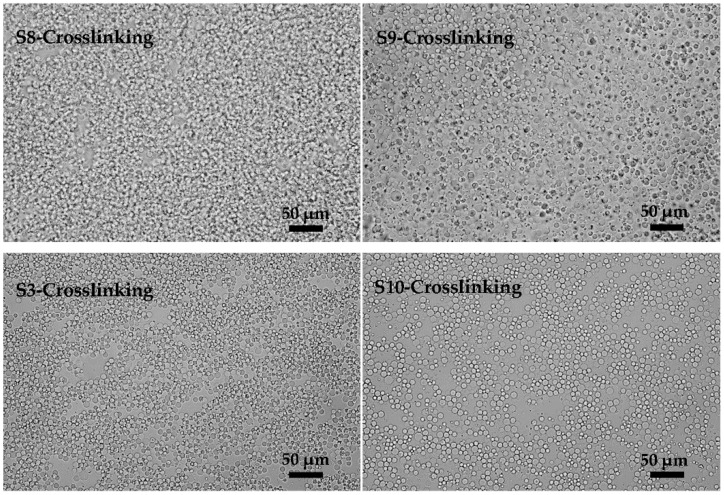
Microscopic photographs of KGM microspheres prepared with different oil phase compositions (7:5, 10:2, 11:1 and 12:0, LP/EP, *w*/*w*): S8-Crosslinking, S9-Crosslinking, S3-Crosslinking and S10-Crosslinking were the crosslinking results of **S8**, **S9**, **S3** and **S10**.

Since the viscosity of liquid paraffin was much higher than that of petroleum ether, the viscosity of the mixed oil phase decreased with the increase of the amount of petroleum ether (shown in Figure 8). In Figure 9 (S8-Crosslinking, S9-Crosslinking, S3-Crosslinking, and S10-Crosslinking), the crosslinking results with different oil phase composition are shown. Serious flocculation occurred in Figure 9 (S8-Crosslinking) due to the low viscosity of the oil phase. By increasing the viscosity of the oil phase, flocculation was weakened (Figure 9 (S9-Crosslinking)). As the viscosity of the oil phase was further increased, the dispersity of the final microspheres became better (Figure 9 (S3-Crosslinking and S10-Crosslinking)). When droplets approached each other, the discharging rate of the liquid between the deformed droplets partly depended on the viscosity of the continuous phase. The greater the viscosity was, the lower the liquid discharging rate and the flocculation rate were [31]. Considering the effects of oil phase composition on emulsification and crosslinking results, the optimum oil phase composition was 11:1 (*w*/*w*, LP/EP).

### 3.4. Effects of the Feeding Methods of EC on the Preparation of KGM Microspheres

**Figure 10:**
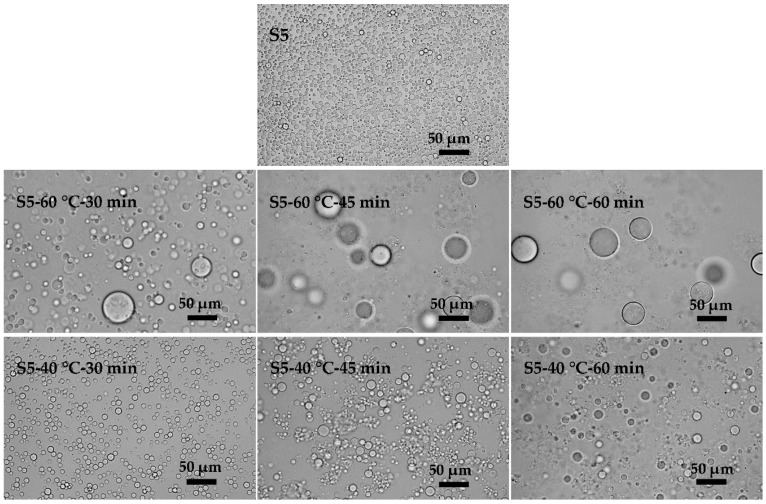
Microscopic photographs of KGM emulsions for **S5**. After emulsification, emulsions of **S5** were kept at 60 °C (or 40 °C) for 30, 45 and 60 min with stirring.

**Figure 11:**
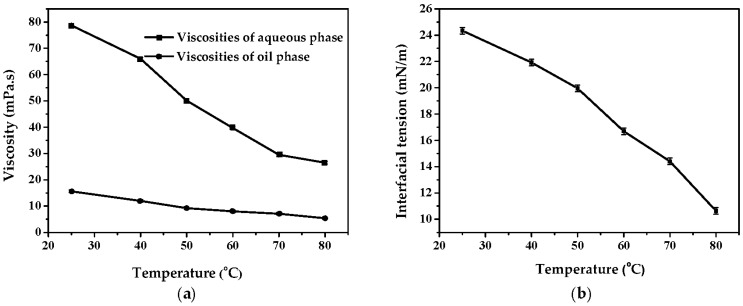
The viscosities of oil (LP/EP, 11/1 (*w*/*w*)) and aqueous phases (KGM I) and the interfacial tension between oil phase and aqueous phases at different temperatures. (**a**) Viscosities of oil and aqueous phases at different temperatures; (**b**) Interfacial tensions between oil and aqueous phases at different temperatures.

In the crosslinking process, flocculation and coalescence occurred frequently. Figure 10 shows the evolution of emulsions with time in the absence of a crosslinking agent. Flocculation and aggregation occurred at different times in the emulsions with different stabilities. The results indicated that low temperature contributed to the stability of the emulsions. An increase in the temperature increased the solubility of the emulsifier in the oil and aqueous phases, so that part of the emulsifier would be desorbed from the oil-water interface, leading to the reduction of the interfacial film strength and the decline of the emulsion stability [32]. Meanwhile, the viscosities of the oil phase and aqueous phase increased with temperature (Figure 11a). The collision energy between the emulsion droplets would decrease for the high motion resistance resulting from the high viscosity of the oil phase. The high viscosity of the aqueous phase could partly inhibit flocculation due to the high viscoelasticity. In addition, high temperature would promote the Brown movement and increase the chance of collision between droplets which would induce the flocculation and coalescence of the emulsions. Although the interfacial tension got larger at low temperature (Figure 11b), it played a minor role. Temperature had an important influence on the stability of the emulsions. 

**Figure 12:**
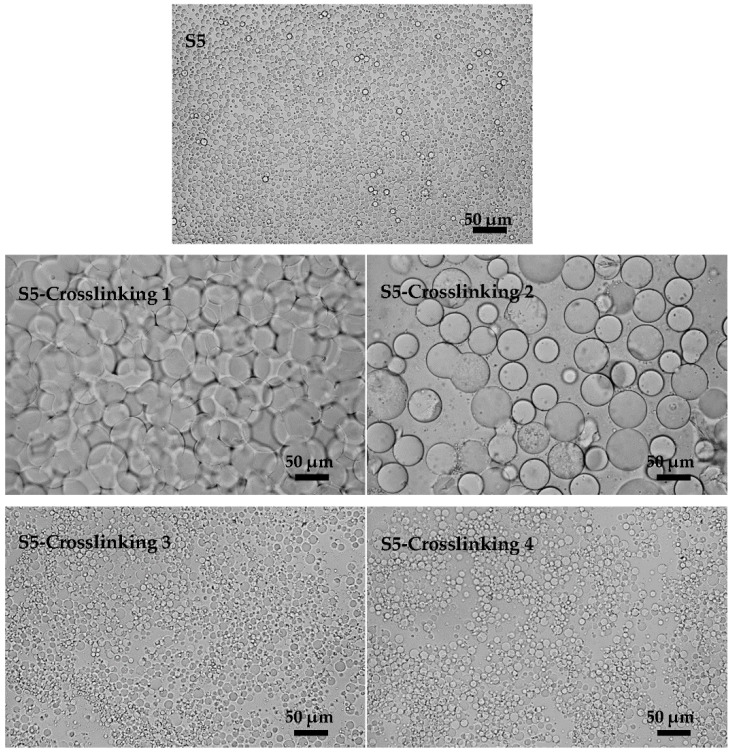
Microscopic photographs of KGM microspheres prepared by different feeding modes of EC: **S5** was KGM emulsions before crosslinking and S5-Crosslinking 1 (EC was added slowly at 60 °C), S5-Crosslinking 2 (emulsions were stirred at 60 °C for 1 h and then EC was added), S5-Crosslinking 3 (EC solution (20 wt %, EC was mixed with oil phase adequately) was added in one batch at 60 °C) and S5-Crosslinking 4 (EC was added at 40 °C and then the temperature was increased to 60 °C) were the crosslinking results of **S5**.

Before coalescence and flocculation occurred, emulsions generally kept stable for a period. During this period, increasing the crosslinking rate would partly inhibit the coalescence and flocculation. So it was very important to increase the crosslinking rate at the beginning of the crosslinking process. Here, we adjusted the crosslinking rate by changing the feeding method of the crosslinking agent (EC). In the traditional method, the EC was added slowly at the crosslinking temperature (60 °C). Here, two different methods were proposed. In the first one, EC was added at 40 °C and then the temperature was increased gradually to 60 °C. Sufficient dispersion of EC in the oil phase could be achieved at 40 °C. During this period the emulsions were in stable state because that low temperature contributed to emulsion stability. As the crosslinking temperature (60 °C) was reached, the well-dispersed EC would react more rapidly with the emulsion droplets. In the second one, EC solution (20 wt %, EC was mixed with oil phase adequately) was added in one batch at the reaction temperature (60 °C). It was easier for the EC solution to spread to the microsphere surface. It can be seen from the results in Figure 12 that flocculation and coalescence occurred in S5-Crosslinking 1 which was cured by the traditional method. In S5-Crosslinking 2, emulsions were stirred at 60 °C for 1 h after emulsification and then EC was added. In this process, emulsion coalescence was almost fully developed before crosslinking and the new emulsions were in stable state, so the obtained microspheres had better dispersity than those in S5-Crosslinking 1. Both of the methods (Figure 12 (S5-Crosslinking 3 and S5-Crosslinking 4)) improved the crosslinking results. The final microspheres prepared with the two different feeding methods had better dispersity and the coalescence and flocculation were effectively suppressed.

## 4. Conclusions

Uniform small-sized KGM microspheres were successfully prepared with the EP membrane we developed by premix membrane emulsification. The stability of emulsions had an important influence on the uniformity and dispersity of the final KGM microspheres. It was found that the preparation condition of the emulsifier 5 wt % PO-5s, KGM III (145.6 mPa·s), and weight ratio of LP to PE 11:1 was in favor of the uniformity and dispersity of the final KGM microspheres. The addition of emulsifier was beneficial to the stability of emulsions, but the excess emulsifier would increase the hydrophilicity of the membrane and produce large droplets. High viscosity of the KGM solution could partly improve the uniformity and stability of emulsions and the greater the oil phase viscosity was, the better the emulsion stability was. In addition, the feeding methods of EC played a significant role in the crosslinking results. Different from the traditional feeding method of EC, the two feeding methods proposed in this study could partly inhibit the coalescence and flocculation, which effectively improved the crosslinking results.

## Figures and Tables

**Table 1:** Konjac glucomanna (KGM) solutions with different viscosities.

Samples	KGM I	KGM II	KGM III	KGM IV
Viscosity (mPa·s) ^a^	66.0 ± 0.5	88.4 ± 0.5	145.6 ± 0.5	180.3 ± 0.5

^a^ All of data were the average values from three determinations.

**Table 2:** Recipe for preparing KGM microspheres.

Sample	Emulsifier Concentration in Oil Phase (*w*/*w*)	KGM Solution	Weight Ratio of Liquid Paraffin (LP) and Petroleum Ether (PE) (*w*/*w*)
**S1**	3%	KGM III	11:1
**S2**	4%	KGM III	11:1
**S3**	5%	KGM III	11:1
**S4**	6%	KGM III	11:1
**S5**	5%	KGM I	11:1
**S6**	5%	KGM II	11:1
**S7**	5%	KGM IV	11:1
**S8**	5%	KGM III	7:5
**S9**	5%	KGM III	10:2
**S10**	5%	KGM III	12:0

## Abbreviations

The following abbreviations are used in this manuscript:

EP: Epoxy-based Polymer membrane
KGM: Konjac Glucomannan
SPG: Shirasu Porous Glass membrane
LP: Liquid Paraffin
PE: Petroleum Ester
CV: Coefficient of Variation
EC: Epoxy Chloropropane
Po-5s: Hexagly Cerinpenta Ester

## References

[1] Vanderbeek P.B., Fasano C., O’Malley G., Hornstein J. (2007). Esophageal obstruction from a hygroscopic pharmacobezoar containing glucomannan. Clin. Toxicol..

[2] Han1A B., Zhang C., Luo X. (2011). Study of konjac glucomannan esterification with dicarboxylic anhydride and effect of degree of esterification on water absorbency. Key Eng. Mater..

[3] Zhang T., Xue Y., Li Z., Wang Y., Xue C. (2015). Effects of deacetylation of konjac glucomannan on alaska pollock surimi gels subjected to high-temperature (120 °C) treatment. Food Hydrocoll..

[4] Wang S., Zhan Y., Wu X., Ye T., Li Y., Wang L., Chen Y., Li B. (2014). Dissolution and rheological behavior of deacetylated konjac glucomannan in urea aqueous solution. Carbohydr. Polymer.

[5] Tian D., Li S., Liu X., Wang J., Liu C. (2013). Synthesis and properties of konjac glucomannan-*graft*-poly(acrylic acid-*co*-trimethylallyl ammonium chloride) as a novel polyampholytic superabsorbent. Adv. Polym. Technol..

[6] Shen C., Li W., Zhang L., Wan C., Gao S. (2012). Synthesis of cyanoethyl konjac glucomannan and its liquid crystalline behavior in an ionic liquid. J. Polym. Res..

[7] Xiong Z.D., Zhou W.Q., Sun L.J., Li X.N., Zhao D.W., Chen Y., Li J., Ma G.H., Su Z.G. (2014). Konjac glucomannan microspheres for low-cost desalting of protein solution. Carbohydr. Polym..

[8] Wang J. (2010). Study on konjac glucomannan accumulation in cell suspension culture of *Amorphophallus konjac*. J. Anhui Agric. Sci..

[9] Guo X.M., Wang G.L., Wei-Lin Y.E., Zhou Z.X., Xiang Y., Zhu X. (2008). Hydrogen peroxide biosensor based on immobilizing enzyme with deacetyled konjac glucomannan. J. Instrum. Anal..

[10] Chen L.G., Liu Z.L., Zhuo R.X. (2005). Synthesis and properties of degradable hydrogels of konjac glucomannan grafted acrylic acid for colon-specific drug delivery. Polymer.

[11] Korkiatithaweechai S., Umsarika P., Praphairaksit N., Muangsin N. (2011). Controlled release of diclofenac from matrix polymer of chitosan and oxidized konjac glucomannan. Marine Drugs.

[12] Ma G.H., Su Z.G., Wang J.X., Ge J.L. (2010). A Konjac Glucomannan Microsphere and Its Preparation Method.

[13] Shen J. (2012). A Method to Prepare Konjac Glucomanan Microspheres.

[14] Nakashima T., Shimizu M., Kukizaki M., Nakashima T., Shimizu M., Kukizaki M. (1992). Membrane emulsification by microporous glass. Key Eng. Mater..

[15] Piacentini E., Drioli E., Giorno L. (2014). Membrane emulsification technology: Twenty-five years of inventions and research through patent survey. J. Membr. Sci..

[16] Van der Graaf S., Schroen C., Boom R.M. (2005). Preparation of double emulsions by membrane emulsification—A review. J. Membr. Sci..

[17] Zhang Y.Q., Xie B.J., Gan X. (2005). Advance in the applications of konjac glucomannan and its derivatives. Carbohydr. Polym..

[18] Charcosset C., Limayem I., Fessi H. (2004). The membrane emulsification process—A review. J. Chem. Technol. Biotechnol..

[19] Kohler J., Chase D.B., Farlee R.D., Vega A.J., Kirkland J.J. (1986). Comprehensive characterization of some silica-based stationary phases for high-performance liquid chromatofraphy. J. Chromatogr..

[20] Mi Y., Zhou W., Li Q., Gong F., Zhang R., Ma G., Su Z. (2015). Preparation of water-in-oil emulsions using a hydrophobic polymer membrane with 3D bicontinuous skeleton structure. J. Membr. Sci..

[21] Vladisavljevic G.T., Kobayashi I., Nakajima M., Williams R.A., Shimizu M., Nakashima T. (2007). Shirasu porous glass membrane emulsification: Characterisation of membrane structure by high-resolution X-ray microtomography and microscopic observation of droplet formation in real time. J. Membr. Sci..

[22] Bardenhagen I., Dreher W., Fenske D., Wittstock A., Bäumer M. (2014). Fluid distribution and pore wettability of monolithic carbon xerogels measured by ^1^H NMR relaxation. Carbon.

[23] Mi Y., Zhou W.Q., Li Q., Zhang D.L., Zhang R.Y., Ma G.H., Su Z.G. (2015). Detailed exploration of structure formation of an epoxy-based monolith with three-dimensional bicontinuous structure. RSC Adv..

[24] Miyagawa Y., Katsuki K., Matsuno R., Adachi S. (2015). Effect of oil droplet size on activation energy for coalescence of oil droplets in an *o*/*w* emulsion. Biosci. Biotechnol. Biochem..

[25] Becher P. (1983). Encyclopedia of Emulsion Technology, Vol. 1: Basic Theory.

[26] Kabalnov A.S., Shchukin E.D. (1992). Ostwald ripening theory—Applications to fluorocarbon emulsion stability. Adv. Colloid Interface Sci..

[27] Schröder V., Behrend O., Schubert H. (1998). Effect of dynamic interfacial tension on the emulsification process using microporous, ceramic membranes. J. Colloid Interface Sci..

[28] Zhou Q., Wang L., Ma G., Su Z. (2008). Multi-stage premix membrane emulsification for preparation of agarose microbeads with uniform size. J. Membr. Sci..

[29] Silva H.D., Cerqueira M.A., Vicente A.A. (2015). Influence of surfactant and processing conditions in the stability of oil-in-water nanoemulsions. J. Food Eng..

[30] Wasan D.T. (1992). Interfacial Transport Processes and Rheology.

[31] Sjöblom J. (2006). Emulsions and Emulsion Stability.

[32] Wang X., Brandvik A., Alvarado V. (2010). Probing interfacial water-in-crude oil emulsion stability controls using electrorheology. Energy Fuels.

